# Tibial bone transport using external fixation in adults: a systematic review

**DOI:** 10.1007/s00402-026-06438-6

**Published:** 2026-08-11

**Authors:** Cristina Giuli, Giulia Di Pietro, Francesco Farine, Maria Beatrice Bocchi, Raffaele Vitiello, Osvaldo Palmacci

**Affiliations:** 1 Catholic University of the Sacred Heart, Rome, Italy; 2 Agostino Gemelli University Polyclinic, Rome, Italy

**Keywords:** Bone transport, Tibia, Ilizarov external fixation, Outcomes, Preoperative comorbidities, Complications

## Abstract

**Introduction:**

Tibial bone transport using external fixation is widely employed for the reconstruction of large and complex tibial bone defects; however, indications and surgical strategies remain highly heterogeneous. Tibial bone transport using external fixation is a demanding but reliable reconstructive technique, characterized by low failure rates and generally favorable outcomes despite frequent complications. Adequate soft-tissue management, selective docking-site grafting, and early functional rehabilitation were commonly reported. Further prospective studies are needed to standardize treatment strategies and improve clinical decision-making.

**Materials and Methods:**

A systematic review was conducted in accordance with PRISMA guidelines in April 2025, searching PubMed/MEDLINE and the Cochrane Library. Extracted data included patient demographics, comorbidities, indications, defect length, surgical technique, frame type, external fixation time, healing index, complications, failures, and ASAMI bone and functional outcomes. Methodological quality was assessed using the MINORS score and results were synthesized descriptively.

**Results:**

A total of 3543 patients from 89 studies were included. Post-traumatic infection or osteomyelitis accounted for 58.5% of indications. The mean defect length was 7.04 cm. Bifocal bone transport was performed in 82.4% of cases, and circular external fixation was used in 79.0%. Early weight-bearing was allowed in approximately 95% of patients. The mean external fixation time was 11.0 months, with a healing index of 54.2 days/cm. The overall failure rate was 25/2748 (0.9%). Most studies were Level III–IV and were of fair-to-good methodological quality.

**Conclusion:**

Tibial bone transport using external fixation is a demanding but reliable reconstructive technique, characterized by low failure rates andgenerally favorable outcomes despite frequent complications. Adequate soft-tissue management, selective docking-site grafting, and early functional rehabilitation were commonlyreported. Further prospective studies are needed to standardize treatment strategies and improve clinical decision-making.

**Level of Evidence:**

III-IV

## Introduction

Reconstruction of large bone defects resulting from acute trauma, chronic osteomyelitis, or tumor resection remains one of the most challenging problems in orthopedic surgery. Historically, primary amputation was often considered the treatment of choice, particularly in fractures associated with extensive bone loss [1]. Over the past decades, however, the development of advanced reconstructive techniques has enabled limb salvage strategies aimed at restoring bone continuity while preserving limb length and function.

Current treatment options for large bone defects include autologous bone grafting for defects smaller than 5 cm, the Masquelet induced membrane technique, acute shortening followed by gradual lengthening, and bone transport using external fixation based on the Ilizarov principles [1–4]. Among these strategies, bone transport through distraction osteogenesis has gained widespread acceptance, particularly in the management of complex, infected, and post-traumatic defects, owing to its ability to regenerate bone and surrounding soft tissues simultaneously.

The Ilizarov method, introduced in the 1950s by Gavril Abramovich Ilizarov in the Soviet Union [5], is based on the principle of controlled mechanical distraction to stimulate new bone formation. Distraction osteogenesis consists of four distinct phases: osteotomy, latency, distraction, and consolidation. Optimal outcomes have been reported when a low-energy corticotomy is followed by a latency period of 5–7 days, gradual distraction at approximately 1 mm per day, and stable external fixation [6]. Using this technique, bone segments of up to 20 cm can be successfully regenerated.

The Ilizarov apparatus is a circular external fixator composed of rings connected by rods and tensioned wires. Frame stability represents a key determinant of successful outcomes and can be optimized through appropriate ring sizing, wire configuration, and construct rigidity [7–9]. Despite its biological advantages and versatility, bone transport using external fixation remains a technically demanding procedure and is associated with a broad spectrum of treatment-related difficulties.

In 1990, Paley et al. proposed a classification system distinguishing problems, obstacles, and complications encountered during limb reconstruction [10]. This framework underscores that many adverse events are intrinsic to the bone transport process and vary in severity and clinical relevance. Commonly reported issues include muscle contractures, joint stiffness, axial deviations, delayed consolidation, nonunion, pin-tract infections, and hardware-related complications.

A representative clinical example involved a 55-year-old male who developed an infected tibial pseudoarthrosis following a Gustilo–Anderson type IIIA open fracture, complicated by a soft-tissue defect requiring coverage with a skin graft. Bone transport was, subsequently, performed to address a 4-cm bone defect. The external fixation time was 8 months, corresponding to a healing index of 60 days/cm, with a follow-up of 2 years. Protected weight-bearing with crutches was allowed starting one month postoperatively, and systemic antibiotic therapy was continued until one month after circular external fixator application. Relevant comorbidities included type II diabetes mellitus complicated by peripheral arterial disease. During treatment, no complications were observed at the docking site, although delayed regenerate formation occurred and progressively improved without the need for additional surgical procedures. The patient developed a deep vein thrombosis, which was successfully managed with anticoagulant therapy, as well as transient paresthesia involving the third, fourth, and fifth toes of the right foot. A mild axial deviation (< 5°) was corrected through fixator adjustments. No pin-tract infections were reported. Following fixator removal, a protective cast was applied for one month, after which the patient resumed progressive full weight-bearing. At final follow-up, no residual sequelae were observed, and both clinical and radiographic outcomes were considered satisfactory.

Despite extensive clinical experience with tibial bone transport, the available literature remains highly heterogeneous with regard to reported outcomes, complication rates, and treatment strategies. A comprehensive synthesis of the evidence is therefore required to better define indications, expected results, and complication profiles associated with this technique.

The aim of this systematic review was to analyze the current literature on tibial bone transport using external fixation in adult patients, focusing on patient characteristics, defect etiology, surgical strategies, treatment duration, complication profiles, and clinical outcomes. The ultimate goal was to provide clinically relevant information to support surgical decision-making and to identify variables suitable for future meta-analytic investigations.

## Materials and methods

### Search strategy

A systematic review of the literature was conducted in accordance with the Preferred Reporting Items for Systematic Reviews and Meta-Analyses (PRISMA 2020) guidelines in April 2025 [11]. The analysis was performed using PubMed/MEDLINE and the Cochrane Library databases to identify studies reporting outcomes of tibial bone transport performed with external fixation. Although the review protocol was not prospectively registered in PROSPERO (International prospective register of systematic reviews), the study protocol, eligibility criteria, and outcomes were predefined before data extraction. No methodological changes were made during the review process.

The search strategy combined the following keywords: “bone transport” AND “tibia” AND “Ilizarov external fixation”. No database filters were applied in order to minimize the risk of missing relevant studies. Additionally, the reference lists of all included articles were manually screened to identify further eligible studies not retrieved through the electronic search. No restrictions were applied regarding the year of publication. The titles of journals, authors’ names, and supporting institutions were not masked at any stage of the review process.

### Eligibility criteria

Studies were included if they:

1. Reported clinical outcomes of tibial bone transport performed using circular or monolateral external fixation;
2. Included only adult patients (≥ 18 years old);
3. Provided extractable data on patient demographics, defect characteristics, surgical strategy, treatment duration, complications, comorbidities or outcomes.

Exclusion criteria were:

1. Case reports with only one patient described,
2. Systematic reviews,
3. Animal or cadaveric studies,
4. Technical notes without clinical outcomes,
5. Non-English language publications.

When studies reported mixed populations (e.g., femoral and tibial defects), only data specifically referring to tibial bone transport were included when extractable.

### Study selection

Three reviewers independently performed the literature search and screened the retrieved records. Duplicates were removed manually prior to screening. Titles and abstracts were reviewed for all search results, and potentially eligible studies underwent full-text evaluation. Disagreements between reviewers were resolved through discussion, and when consensus could not be reached, the senior author was consulted.

### Data extraction and analysis

Data from the included studies were independently extracted by three authors and entered into a standardized Excel spreadsheet. Extracted variables included: first author, year of publication, study design, number of patients, age and sex distribution, etiology of bone defects, comorbidities, defect length, number of osteotomies, type of external fixator, soft-tissue reconstruction performed prior to bone transport, external fixation time (EFT), healing index (HI), duration of follow-up, treatment failures, Paley classification (problems, obstacles, complications), and ASAMI bone and functional scores. The extracted dataset was subsequently reviewed by two authors, who verified data accuracy and resolved discrepancies by consensus. Results were synthesized descriptively.

Given substantial clinical and methodological heterogeneity, no meta-analysis or inferential statistical testing was performed. Continuous variables were summarized as weighted means, weighted by the sample size of each study, with ranges reported as minimum and maximum values. Categorical variables were reported as frequencies and percentages. ASAMI scores were expressed as percentages based on the number of patients for whom these outcomes were available. All analyses were based on the data available in the original studies; outcomes were analyzed only when explicitly reported.

### Methodological quality and risk of bias assessment

Methodological quality was assessed using the Methodological Index for Non-Randomized Studies (MINORS). The MINORS instrument includes 12 items (8 for non-comparative studies and 12 for comparative studies), each scored from 0 to 2, with a maximum total score of 16 for non-comparative studies and 24 for comparative studies [12]. Quality assessment was performed independently by the reviewers following the same process described for study selection and data extraction.

For non-comparative studies, total scores were interpreted as very low quality (0–4), low quality (5–7), fair quality (8–12), and high quality (≥ 13). For comparative studies, total scores were interpreted as very low quality (0–6), low quality (7–10), fair quality (11–15), good quality (16–20), and very good quality (21–24).

Given the absence of a quantitative meta-analysis, funnel plots and statistical tests for publication bias were not performed. To minimize the risk of missing relevant studies, we conducted comprehensive searches in PubMed/MEDLINE and the Cochrane Library and screened the reference lists of included articles to identify additional relevant articles. Nevertheless, publication bias cannot be excluded, particularly given the predominance of retrospective and non-comparative designs.

### Certainty of evidence

Overall certainty of evidence for key outcomes was assessed qualitatively using an approach adapted from GRADE (Grading of recommendations assessment, development and evaluation) principles, considering study design, methodological quality (MINORS), consistency of findings, and completeness of outcome reporting. The certainty of evidence was categorized as high, moderate, low, or very low.

## Results

### Search and literature selection

The initial literature search identified a total of 355 records. After removal of duplicates and screening based on the predefined inclusion and exclusion criteria, 152 articles were selected for full-text review. Studies reporting mixed reconstructive techniques or alternative treatment strategies were excluded to ensure methodological homogeneity. Ultimately, 89 studies met the inclusion criteria and were included in the final analysis.

**Fig. 1:**
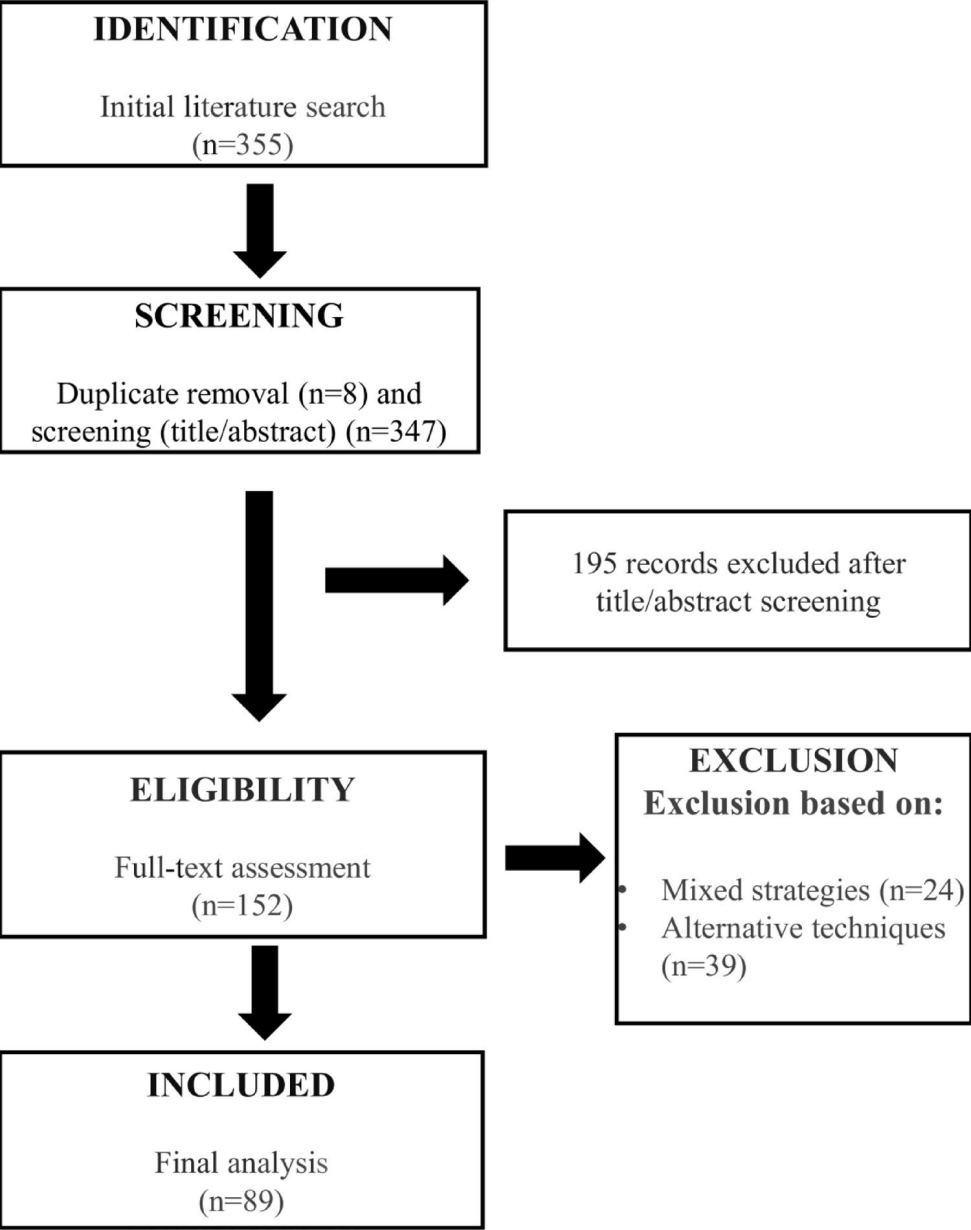
PRISMA flow chart

The study selection process is illustrated in the PRISMA flow chart (Fig. 1).

### Study characteristics

A total of 89 studies were included, comprising 78 retrospective studies, 9 prospective studies, and 2 case series. According to the Oxford Centre for Evidence-Based Medicine (OCEBM) criteria, 4 studies were Level II, 36 were Level III, and 49 were Level IV.

Methodological quality was assessed using the Methodological Index for Non-Randomized Studies (MINORS) score according to predefined cut-off values. Among the 40 comparative studies, 1 was rated as very good, 30 as good, and 9 as fair quality. Among the 49 non-comparative studies, 7 were rated as high quality and 40 as fair quality; no studies were classified as low or very low quality. Overall, although most studies were rated as fair-to-good quality, the predominance of retrospective and non-comparative designs suggests an inherent risk of selection bias and confounding.

Detailed study characteristics, including study design, sample size, publication year, MINORS scores, and OCEBM level of evidence, are summarized in Table 1.

**Table 1:** Study characteristics and methodological quality

First author	Year	Study design	Comparative status	No. of patients	MINORS SCORE	Level of evidence
Zhang Y [13]	2025	Retrospective	Comparative	48	20/24	III
Xuming W [14]	2024	Retrospective	Comparative	62	17/24	III
Zheng K [15]	2024	Retrospective	Non-comparative	4	14/16	IV
Kummer A [16]	2024	Retrospective	Comparative	24	18/24	III
Liu K [17]	2023	Retrospective	Non-comparative	62	14/16	IV
Yang J [18]	2024	Retrospective	Non-comparative	363	11/16	IV
Shi B [19]	2024	Retrospective	Comparative	62	16/24	III
Zhang Q [20]	2024	Retrospective	Comparative	30	18/24	III
Feng D [21]	2023	Retrospective	Non-comparative	103	12/16	IV
Wang Q [22]	2024	Retrospective	Comparative	35	20/24	III
Kukreja K [23]	2024	Prospective	Comparative	49	17/24	II
Shastov AL [24]	2023	Retrospective	Comparative	6	17/24	III
Hamiti Y [25]	2024	Retrospective	Comparative	20	19/24	III
Sheridan GA [26]	2023	Retrospective	Comparative	90	18/24	III
Cao ZM [27]	2023	Retrospective	Comparative	35	18/24	III
Cao Z [28]	2022	Retrospective	Non-comparative	72	12/16	IV
Liu K [29]	2022	Retrospective	Comparative	21	17/24	III
Abulaiti A [30]	2022	Retrospective	Comparative	53	17/24	III
Yushan M [31]	2022	Retrospective	Comparative	12	18/24	III
Rohilla R [32]	2021	Prospective	Comparative	13	18/24	II
Kliushin NM [33]	2021	Retrospective	Comparative	31	17/24	III
Xu YQ [34]	2021	Retrospective	Non-comparative	31	10/16	IV
Hamiti Y [35]	2022	Retrospective	Comparative	18	17/24	III
Hamiti Y [36]	2021	Retrospective	Non-comparative	18	10/16	IV
Li Y [37]	2021	Retrospective	Non-comparative	12	10/16	IV
Liu K [38]	2021	Retrospective	Non-comparative	236	8/16	IV
Feng D [39]	2023	Retrospective	Non-comparative	199	10/16	IV
Huang Q [40]	2021	Retrospective	Comparative	41	16/24	III
Miraj F [41]	2021	Retrospective case series	Non-comparative	14	11/16	IV
Yalikun A [42]	2021	Retrospective	Non-comparative	149	11/16	IV
Li R [43]	2021	Retrospective	Non-comparative	40	11/16	IV
Baruah RK [44]	2020	Retrospective	Comparative	40	14/24	III
Ren GH [45]	2020	Retrospective	Comparative	32	16/24	III
Jilani LZ [46]	2020	Prospective	Comparative	8	18/24	II
Carlo Biz [47]	2020	Retrospective	Comparative	56	17/24	III
Jitendra Wadhwani [48]	2020	Retrospective	Comparative	35	17/24	III
Abulaiti Abula [49]	2020	Retrospective	Comparative	14	18/24	III
Runguang Li [50]	2020	Retrospective	Non-comparative	68	11/16	IV
Henrique Carvalho de Resende [51]	2020	Retrospective	Non-comparative	22	10/16	IV
Yang Li [52]	2020	Retrospective	Comparative	26	18/24	III
Sigmund IK [53]	2020	Prospective	Comparative	27	18/24	II
Yushan M [54]	2020	Retrospective	Comparative	37	19/24	III
Hakan Kinik [55]	2021	Retrospective	Non-comparative	30	10/16	IV
Mootaz F Thakeb [56]	2019	Prospective	Comparative	25	22/24	II
Fahad S [57]	2019	Retrospective	Non-comparative	51	10/16	IV
Ayush Kumar Singh [58]	2019	Prospective	Non-comparative	27	10/16	IV
Karim Bakhsh [59]	2019	Case series	Non-comparative	56	10/16	IV
Catagni MA [60]	2019	Retrospective	Comparative	86	18/24	III
Bhowmick K [61]	2019	Retrospective	Non-comparative	8	10/16	IV
Dmitry Y. Borzunov [62]	2017	Retrospective	Non-comparative	7	8/16	IV
Yongwei Wu [63]	2017	Retrospective	Comparative	23	17/24	III
Tong K [64]	2017	Retrospective	Comparative	13	16/24	III
Aktuglu K [65]	2016	Retrospective	Non-comparative	24	11/16	IV
Abuomira IE [66]	2016	Retrospective	Comparative	55	14/24	III
Sadek AF [67]	2016	Retrospective	Comparative	14	15/24	III
Fürmetz J [68]	2016	Retrospective	Comparative	25	13/24	III
Repo JP [69]	2016	Retrospective	Comparative	11	13/24	III
Yin P [70]	2015	Retrospective	Non-comparative	72	9/16	IV
Azzam W [71]	2015	Retrospective	Non-comparative	30	11/16	IV
Wani NB [72]	2015	Retrospective	Non-comparative	18	9/16	IV
Lakhani A [73]	2014	Retrospective	Non-comparative	19	9/16	IV
Harshwal RK [74]	2014	Retrospective	Non-comparative	5	13/16	IV
Mora R [75]	2014	Retrospective	Non-comparative	120	11/16	IV
Karargyris O [76]	2013	Retrospective	Non-comparative	7	11/16	IV
Spiegl U [77]	2013	Prospective	Non-comparative	25	13/16	IV
Atef A [78]	2013	Retrospective	Comparative	15	20/24	III
Lowenberg D [79]	2013	Retrospective	Non-comparative	34	13/16	IV
Lovisetti G [80]	2013	Retrospective	Comparative	39	20/24	III
Yusof NM [81]	2012	Retrospective	Non-comparative	4	13/16	IV
Sala F [82]	2011	Retrospective	Comparative	12	19/24	III
Chaddha M [83]	2010	Retrospective	Non-comparative	22	13/16	IV
Iacobellis C [84]	2010	Retrospective	Comparative	74	16/24	III
Bumbasirevic M [85]	2010	Retrospective	Non-comparative	30	13/16	IV
Megas P [86]	2010	Retrospective	Non-comparative	9	10/16	IV
Emara KM [87]	2008	Prospective	Comparative	16	19/24	II
Madhusudhan TR [88]	2008	Prospective	Non-comparative	9	11/16	IV
El-Gammal TA [89]	2008	Retrospective	Comparative	12	17/24	III
Farmanullah [90]	2007	Retrospective	Non-comparative	58	9/16	IV
Brinker MR [91]	2007	Prospective	Non-comparative	2	11/16	IV
Fabry K [92]	2006	Retrospective	Non-comparative	10	9/16	IV
Rozbruch SR [93]	2006	Retrospective	Non-comparative	15	10/16	IV
Mahaluxmivala J [94]	2005	Retrospective	Comparative	6	17/24	III
McHale KA [95]	2004	Retrospective	Non-comparative	5	9/16	IV
Mekhail AO [96]	2004	Retrospective	Non-comparative	16	10/16	IV
Thirumal M [97]	2001	Retrospective	Non-comparative	21	10/16	IV
Duman H [98]	2001	Retrospective	Non-comparative	9	9/16	IV
Yokoyama K [99]	2001	Retrospective	Comparative	4	13/24	III
Song HR [100]	1998	Retrospective	Non-comparative	27	9/16	IV
Polyzois D [101]	1997	Retrospective	Non-comparative	25	10/16	IV

Comparative studies were scored out of 24 and non-comparative studies out of 16 (MINORS). Level of evidence assigned according to OCEBM criteria (II: prospective comparative; III: retrospective comparative; IV: non-comparative case series/cohorts)

### Demographic characteristics and comorbidities

Overall, the included studies involved a total of 3543 adult patients undergoing tibial bone transport with external fixation. The weighted mean age across patients was 38.24 years, the range refers to the minimum and maximum values reported across included studies (9.5–71 years). Sex was reported for 3032 patients: most were male (2,413; 79.6%), while 619 (20.4%) patients were female.

Preoperative comorbidities were not reported in all studies. Therefore, percentages for each comorbidity were calculated only among patients from studies that reported that specific condition, and not on the total cohort of 3543 patients. Consequently, the number of patients considered for each comorbidity was always lower than the overall study population.

Among the reported data, obesity was reported in 337/1343 patients (25.1%), smoking in 228/1036 (22.0%), diabetes mellitus in 182/1636 (11.1%), chronic glucocorticoid therapy in 116/1240 (9.4%), hypertension in 91/1218 (7.5%), osteoporosis in 97/1358 (7.1%), alcohol abuse in 42/1045 (4.0%), and other comorbidities in 45/1339 (3.4%). No study reported the presence of cardiopathy. Sociodemographic characteristics, including educational level, living environment, and occupational status, were inconsistently documented and therefore could not be systematically analyzed.

### Bone transport characteristics and duration of treatment

Etiology was reported for 3519 patients. The most common indication for tibial bone transport was post-traumatic infection or osteomyelitis, accounting for 2057 patients (58.5%), followed by post-traumatic bone defects in 1400 patients (39.8%). Oncological indications were uncommon (16 patients; 0.5%), while other etiologies accounted for 46 patients (1.3%) of cases. The weighted mean bone defect length across patients (weighted by study sample size) was 7.04 cm (range across studies: 1.2–14.5 cm).

Surgical technique and type of external fixation were reported for 3543 patients. Bifocal bone transport was the most frequently adopted technique in 2920/3543 patients (82.4%), followed by trifocal transport in 598/3543 patients (16.9%). Tetrafocal and pentafocal techniques were rarely reported (9/3543 patients, 0.3% and 13/3543 patients, 0.4%, respectively). In a very small proportion of cases (3/3543 patients; 0.1%), the transport technique could not be classified because the number of osteotomy levels was not specified in the original studies.

Circular external fixation was employed in 2799/3543 patients (79.0%), whereas 571/3543 patients (16.1%) were treated using monolateral external fixators. Taylor Spatial Frame constructs were used in 60/3543 patients (1.7%), hybrid fixation was used in 20/3543 patients (0.6%), and the fixation method was not specified in 93/3543 patients (2.6%). Early weight-bearing during treatment was allowed in approximately 95% of cases.

The weighted mean external fixation time across patients (weighted by study sample size) was 11.0 months. The weighted mean healing index across patients was 54.2 days/cm. The weighted mean follow-up duration across patients was 31.3 months (range across studies: 3.7–259 months).

Patient characteristics, causes of bone transport, bone transport technique, and duration of treatment are summarized in Table 2.

**Table 2:** Patient characteristics, causes of bone transport, bone transport technique, and duration of treatment

Category	Variable	Value
Patient characteristics	Mean age (years)	38.24
	Sex (Male/Female)	79.6%/20.4%
Causes of bone transport	Osteomyelitis/post-traumatic infection	58.5%
	Post-traumatic bone defect	39.8%
Bone transport technique	Mean bone defect length (cm)	7.04
	Bifocal transport	82.4%
	Circular external fixator	79.0%
Duration of treatment	External fixation time (months)	11.0
	Healing index (days/cm)	54.2
	Follow-up (months)	31.3

Sex, etiology, and continuous variables reported in Table 2 (defect length, external fixation time, healing index, and follow-up) were analyzed using available data only; therefore, denominators may vary across variables

### Soft tissue reconstruction prior to surgery

Soft-tissue reconstruction prior to the initiation of bone transport was reported in 61 of the 89 studies analyzed. A total of 620 flap procedures and 103 skin grafts were performed before bone transport, as reported only by the studies that documented pre–bone transport soft-tissue procedures, highlighting the frequent need for adequate soft-tissue management in complex tibial reconstructions.

### Complications during bone transport and sequelae

According to the Paley classification, a total of 1775 events were categorized as problems, 1069 as obstacles, and 398 as complications, based exclusively on studies that explicitly reported this classification.

Paley classification data were extracted as event-level counts (problems, obstacles, and complications). When studies additionally reported specific complications at the patient level, we reported the number of affected patients and calculated percentages using outcome-specific denominators.

At the patient level, pin-tract infection was the most frequently reported problem, occurring in 977 patients (45.2%), followed by joint stiffness (349 patients; 23.5%), muscle contracture (151 patients; 19.0%), and axial deviation (359 patients; 18.4%). Percentages were calculated exclusively from the subset of studies reporting problem-specific data.

Reported obstacles included docking-site bone grafting in 423 patients (19.9%), soft-tissue interposition in 167 patients (12.4%), nonunion in 232 patients (11.8%), delayed union in 169 patients (11.4%), pin loosening in 134 patients (10.2%), regenerate-related issues in 94 patients (8.3%), other soft-tissue conditions (e.g., flap failure, skin necrosis) in 59 patients (7.8%), and deep vein thrombosis in 3/36 patients (8.3%). All obstacle-related percentages were calculated only from studies that reported data on these outcomes.

Complications, both minor and major, included limb shortening in 102 patients (11.8%), equinus or equinovarus deformity in 42 patients (9.1%), refracture in 67 patients (4.8%), recurrence of deep infection in 64 patients (4.7%). All complications-related percentages were calculated only from studies that reported data on these outcomes. Complication data according to Paley are summarized in Table 3.

**Table 3:** Complications according to Paley

Paley category	Event	Patients, *n* (%)
Problem	Pin tract infection	977/2162 (45.2%)
Problem	Joint stiffness	349/1483 (23.5%)
Problem	Muscle contracture	151/793 (19.0%)
Problem	Axial Deviation	359/1954 (18.4%)
Obstacle	Soft Tissue Interposition	167/1348 (12.4%)
Obstacle	Nonunion	232/1976 (11.8%)
Obstacle	Delayed union	169/1480 (11.4%)
Obstacle	Pin loosening	134/1314 (10.2%)
Obstacle	Malalignment, delayed ossification or re-fracture of callus distraction zone	94/1136 (8.3%)
Complication	Limb shortening	102/868 (11.8%)
Complication	Equinus/Equinovarus	42/461 (9.1%)
Complication	Refracture	67/1405 (4.8%)
Complication	Deep infections recurrence	64/1374 (4.7%)

Complications reported in Table 3 were analyzed using available data only; therefore,denominators may vary across variables

The overall failure rate, among reports where this variable was available, was low, with 25/2748 reported failures (0.9%), predominantly attributable to uncontrolled primary or recurrent infection and vascular disorders leading to amputation.

### ASAMI score

Functional outcomes assessed using the ASAMI score were available for 2055 patients: outcomes were rated as excellent in 977 patients (47.5%), good in 752 (36.6%), fair in 246 (12.0%), and poor in 80 (3.9%). The remaining 11 patients had incomplete category-level reporting. Bone outcomes were available for 2062 patients and were rated as excellent in 1199 (58.1%), good in 590 (28.6%), fair in 171 (8.3%), poor in 100 (4.9%), and failure in 2 (0.1%). These percentages were calculated only from studies with available ASAMI data.

### Certainty of evidence

Overall certainty of evidence ranged from low to moderate across outcomes. Certainty was considered moderate for external fixation time, healing index, and failure rates, as these outcomes were derived from large cumulative samples and showed relatively consistent descriptive results. Certainty for complication rates was moderate overall, although heterogeneity in definitions and variability in reported denominators across studies may reduce the precision of some results. Certainty was low to moderate for ASAMI outcomes and comorbidities due to inconsistent reporting and the predominance of retrospective, non-comparative study designs.

## Discussion

This systematic review provides a comprehensive overview of tibial bone transport using external fixation, with a particular focus on patient comorbidities, reported failure rates, and clinical outcomes, aiming to support surgical decision-making and to identify variables suitable for future meta-analytical investigations.

The present analysis suggests that tibial bone transport with external fixation, although technically demanding and frequently associated with treatment-related challenges, is characterized by low reported failure rates and generally favorable bone and functional outcomes across the available literature. These findings support the role of bone transport as a reliable reconstructive option for complex tibial defects when appropriately indicated.

A key finding of this review is that only a limited number of studies have specifically investigated preoperative patient comorbidities, and even fewer have analyzed their impact on bone transport outcomes. In fact, among the studies included in this systematic review that clearly reported exclusion criteria, 26 articles explicitly excluded patients with systemic conditions that could potentially impair bone healing, such as diabetes mellitus, hypertension, coronary artery disease, chronic kidney disease and liver cirrhosis [14, 21, 23, 27, 34, 52, 54, 64, 87], as well as skeletal disorders known to affect bone regeneration, including rickets, osteomalacia, primary or metastatic bone tumors, osteopetrosis, and Paget’s disease [48, 68]. Additional exclusion criteria frequently included autoimmune and hematologic disorders, advanced osteoporosis, neurological, psychiatric, or cognitive impairments, poor patient compliance, severe vascular disease precluding limb salvage, active smoking, and inability to tolerate bone transport [13, 18, 19, 29, 30, 37–40, 42, 47, 50, 58, 74, 77].

Only a small subset of studies assessed the relationship between patient-related factors and clinical outcomes, yielding heterogeneous and often conflicting results. Azzam et al. reported that advanced age and smoking negatively influenced treatment outcomes [71], whereas Atef et al. found no significant association between age or sex and bone transport results [78]. Similarly, Fürmetz et al. observed that age and smoking status did not affect treatment success and suggested that patient comorbidities were primarily associated with prolonged hospitalization rather than treatment failure [68]. Conversely, Liu et al. suggested that preoperative comorbidities and living environment may contribute to a higher rate of complications, particularly pin-tract infections [38]. Nevertheless, the available evidence remains limited, heterogeneous, and inconclusive. These findings highlight a significant gap in the literature and underscore the need for well-designed prospective studies and future meta-analyses to clarify whether patient comorbidities influence bone transport outcomes.

Most of the analyzed studies emphasized that soft-tissue reconstruction, when required, is performed either before or concomitantly with the initiation of bone transport. This finding is consistent with the systematic review by Stoneburner et al. [102] and the study by Abulaiti Abula et al. [30], both of which suggest that adequate soft-tissue management is essential to achieve satisfactory functional outcomes following bone transport.

Regarding the docking site, most studies did not perform routine bone grafting but reserved it for selected cases, typically after failure of conservative measures such as the accordion technique. This approach aligns with the systematic review by Liodakis et al. [103], which reported that routine docking-site grafting may expose up to two-thirds of patients to an unnecessary additional procedure without improving final treatment outcomes.

Considerable variability was observed in the surgical techniques used for bone transport. Bifocal bone transport was the most commonly reported approach, whereas trifocal transport was used less frequently. Importantly, the available literature does not allow definitive conclusions regarding the relationship between defect length and the number of osteotomies performed, as comparable defect sizes were treated using different strategies across studies. For example, Wadhwani et al. reported the use of both bifocal and trifocal bone transport for similar defect lengths [48], highlighting the lack of uniform criteria guiding technique selection. Nevertheless, comparative studies by Catagni et al. [60] and Liu et al. [104] suggest that multilevel or trifocal bone transport is preferentially used for the reconstruction of larger segmental defects. In these studies, trifocal transport was associated with shorter external fixation time and a lower healing index, despite being applied to greater defect lengths. These advantages have been attributed to the presence of two simultaneous distraction osteogenesis sites, which may accelerate bone regeneration and reduce the amount of regenerate required at each osteotomy level. This biological and mechanical advantage appears particularly relevant in critical-sized defects, where single-level transport may delay regenerate maturation, increase docking-site problems, and lead to complications related to prolonged external fixation. Despite these encouraging findings, the heterogeneity of indications across studies highlights the lack of standardized treatment algorithms and underscores the need for further high-quality comparative research to better define the role of defect size in guiding surgical strategy.

Finally, regarding postoperative rehabilitation, approximately 95% of the included studies reported allowing early weight-bearing during bone transport to promote functional recovery. However, other authors advocate delaying loading until radiographic union is achieved. This more conservative approach is driven by concerns regarding regenerate bone quality, frame stability, anatomical location, and infection-related factors, as premature loading may increase the risk of deformity, mechanical failure, or compromised bone healing. Differences in patient selection, fixation constructs, and biological environment likely account for the heterogeneity in postoperative weight-bearing protocols reported in the literature [6, 105].

### Limitations of the article

This systematic review has several limitations.

First, most included studies were retrospective and non-comparative, which carries an inherent risk of selection bias and confounding. In addition, studies differed in design and patient populations, and there was substantial clinical heterogeneity, including differences in defect etiology (infected versus non-infected defects), defect length, surgical strategy (bifocal versus trifocal transport), fixation constructs (circular versus monolateral frames), and weight-bearing protocols. Therefore, our results should be interpreted as descriptive and may not be directly comparable across studies.

Second, data were extracted at the study level rather than at the individual patient level. As a result, we could not reliably assess how patient factors (e.g., comorbidities) or defect characteristics influenced outcomes, nor perform robust subgroup analyses.

Third, several variables were not consistently reported across studies. In particular, complications, Paley classification, comorbidities, and ASAMI outcomes were not uniformly available, and many percentages were calculated using outcome-specific denominators rather than the total cohort. This made it difficult to compare results across studies and prevented a quantitative meta-analysis.

Finally, publication bias cannot be excluded, as studies reporting favorable results may be more likely to be published; in the absence of meta-analysis, formal statistical assessment was not feasible. Moreover, some events were based on small denominators (e.g., deep vein thrombosis), which may make these estimates less reliable.

Overall, conclusions should be interpreted with caution, and higher-quality prospective comparative studies with standardized outcome reporting are needed.

Despite these limitations, recurring patterns identified in this systematic review may support clinical decision-making (Table 4).

**Table 4:** Practical take-home messages from this systematic review

Clinical variables	Evidence from this review	Clinical implication
Indication	Post-traumatic infection/osteomyelitis is the most common indication (58.5%)	Bone transport is frequently used in infection-related tibial defects
Comorbidities	Inconsistent reporting and inconclusive associations with outcomes	Prospective studies and individual patient level meta-analyses are needed
Defect length and technique selection	Weighted mean defect length 7.04 cm; technique choice varied across studies	Defect size may be considered when selecting transport strategy, but current evidence does not define clear thresholds
Soft tissue management	Flaps and skin grafts were frequently performed prior to bone transport	Adequate soft-tissue coverage is commonly achieved before or at the initiation of transport in complex defects
Docking site management	Docking-site bone grafting was required in 19.9% of patients	Reported evidence supports selective rather than routine docking-site intervention
External fixation construct	Circular external fixation was most frequently used (79%)	Circular frames represent the predominant construct in the literature
Weight bearing	Early weight bearing was allowed in approximately 95% of cases	Early functional rehabilitation is commonly reported, although protocols vary
Expected treatment duration	Mean external fixation time 11.0 months; healing index 54.2 days/cm	These figures may assist in patient counseling regarding anticipated treatment duration

## Conclusion

Tibial bone transport with external fixation is a demanding but reliable reconstructive option for large and complex tibial bone defects. Despite prolonged treatment and a high incidence of treatment-related problems and complications, reported failure rates are low and overall bone and functional outcomes are generally favorable.

This systematic review suggests that soft-tissue management is most commonly performed before or at the initiation of bone transport and that docking-site interventions are applied selectively rather than routinely. Early functional rehabilitation, including weight-bearing during treatment, is frequently reported; however, postoperative protocols vary considerably across studies.

The available literature remains highly heterogeneous in terms of indications, surgical strategies, and outcome reporting. Patient-related factors, preoperative comorbidities, and sociodemographic variables were inconsistently documented, limiting the ability to describe these variables and explore their association with outcomes. These limitations, together with the lack of standardized criteria for technique selection based on defect characteristics, support the need for further prospective studies and individual patient-level meta-analyses to optimize treatment strategies and improve clinical decision-making.

## Data Availability

The datasets generated and analyzed during the current study are available from the corresponding author upon reasonable request. No analytic code was used beyond standard spreadsheet calculations.

## Abbreviations

ASAMI: Association for the study and application of the Method of Ilizarov
MINORS: Methodological Index for Non-Randomized Studies
OCEBM: Oxford Centre for Evidence-Based Medicine
GRADE: Grading of recommendations assessment, development and evaluation
PRISMA: Preferred Reporting Items for Systematic Reviews and Meta-Analyses
PROSPERO: International prospective register of systematic reviews
HI: Healing Index
EFT: External Fixation Time
